# Grand Challenges for Positive Psychology: Future Perspectives and Opportunities

**DOI:** 10.3389/fpsyg.2022.833057

**Published:** 2022-05-26

**Authors:** Llewellyn E. van Zyl, Sebastiaan Rothmann

**Affiliations:** ^1^Department of Industrial Engineering and Innovation Sciences, University of Eindhoven, Eindhoven, Netherlands; ^2^Optentia Research Unit, North-West University, Vanderbijlpark, South Africa; ^3^Department of Human Resource Management, University of Twente, Enschede, Netherlands; ^4^Department of Social Psychology, Institut für Psychologie, Goethe University, Frankfurt am Main, Germany

**Keywords:** criticisms of positive psychology, critiques, challenges, opportunities, positive psychology

## Introduction

Positive psychology is one of the fastest-growing sub-disciplines of psychology (Donaldson et al., 2021; Martín-del-Río et al., 2021) and established itself as a genuinely transdisciplinary science (Lomas et al., 2021). With applications ranging from neuroscience to architecture and from climate change to criminology (Greene and Seligman, 2016; Sander et al., 2019), the scientific study of the positive states, -traits, and -behaviors underpinning quality of life has flourished (Bohlmeijer and Westerhof, 2021). This paradigm of studying “what's right” rather than “what's wrong” has also led to the establishment of several focus areas ranging from positive risk management, positive health, and positive coaching (Van Zyl et al., 2020b; Richter et al., 2021) to positive organizational psychology (Donaldson and Ko, 2010), positive artificial intelligence (da Silva, 2020) and positive computing (Jeong et al., 2020). Positive psychology has broadened our understanding of the elements underpinning wellbeing and the factors that undermine them with the collective efforts of academics, journals, professional societies, practitioners, and the public (Ng et al., 2021; Worthington and Van Zyl, 2021). With these collective efforts, positive psychology has given birth to several revolutionary theories, methodologies, frameworks, and approaches to measure, explain and develop the conditions required for individuals to thrive, communities to flourish, and societies to prosper (Lomas et al., 2021). These developments have produced significant insights into the human condition and provided innovative solutions to complex individual-, organizational- and societal problems (Worthington and Van Zyl, 2021).

However, despite its growth and contribution, positive psychology is not without its challenges. Since its formal inception in 2000, many scholars have questioned the unique contribution of the paradigm as well as the validity of the theories, methods, interventions, and philosophy underpinning the discipline (Brown et al., 2014; Goodman et al., 2017; Wong and Roy, 2017; Compton and Hoffman, 2019; Van Zyl, 2019; Yakushko, 2019). This, in turn, has negatively affected positive psychology's scientific credibility and public perception (Van Zyl and Ten Klooster, 2022). However, within these criticisms and critiques lies unique opportunities to channel future growth and development of the discipline. Therefore, a clear and consolidated view of the criticisms and critiques is required to help chart a course for future directions in positive psychology. As such, this paper aims to explore seven of the grand challenges facing positive psychology and attempts to identify possible routes for future research areas.

## Criticisms and Critiques: Grand Challenges for Positive Psychology

We live in an increasingly volatile, uncertain, complex and ambiguous (VUCA) world which poses unique challenges for individuals, organizations, communities and societies (Bhattacharya et al., 2020; Wieners et al., 2021). These challenges stem from increasing social tensions between groups, the strain on the natural ecosystem, rising discontent with capitalism and increased economic volatility (Bhattacharya et al., 2020). This in turn, results in increased perceptions of inequality, poverty, and unemployment which ultimately impacts global prosperity. Traditional approaches to addressing these issues seem futile (Bhattacharya et al., 2020), resulting in a need for more innovative or unique approaches to address such. With the advent of the “Third Wave” in positive psychology, Lomas et al. (2021) called for a concerted effort to address these challenges by focusing on understanding the positive states, traits and behaviors required to enhance human functioning and global prosperity. This call places positive psychology in a unique position to affect global change directly. For the discipline to have a global impact, positive psychology should expand into new areas and domains structured around the unique challenges this VUCA world poses. However, for the discipline to expand, it needs to address both these challenges as well as the major criticisms and critiques posed against it during the last decade. But what are the *current* challenges facing the discipline? Albeit not an exhaustive list, we believe that the main challenges for positive psychology can be summarized in seven broad themes.

**First**, Friedman and Brown (2018) argued that *positive psychology lacks a unifying metatheory that underpins the philosophy of the science and provides a fundamental set of ideas about, on how positive psychological phenomena should be thought researched, and approached*. According to Wallis (2010), metatheories provide a set of philosophical principles required for the development of a discipline through (a) clarifying the purpose of theories underpinning a science, (b) stating what types of theories or methods are needed to advance its development, (c) setting and criticizing criteria for theory development and evaluation and (d) highlighting broad and paradigmatic issues relating to general theory development. Metatheories should also be comprised of ever restrictive grand- and mid-range theories and theoretical models/frameworks explaining a phenomenon (Wallis, 2010). Without a clearly articulated, unifying metatheory, positive psychology will be constrained to componential thinking, which is compounded by a hyper-focus on developing specific states, traits or behaviors (Donaldson et al., 2021). Alternatively, it could also lead to little or no consensus on how core concepts of the discipline should be approached or defined (Wallis, 2010; Gruman et al., 2018).

In their position paper, Seligman and Csikszentmihalyi (2000, p. 5) attempted to provide a meta-theoretical paradigm perspective of positive psychology. They defined positive psychology as: “a science of positive, subjective experience, positive individual traits, and positive institutions [aimed at] improving quality of life and to prevent the pathologies that arise when life is barren or meaningless.” This definition and the approach outlined in their paper did not clarify what constitutes “positivity,” nor did it present a clear purpose of positive theories (Donaldson et al., 2021). Further, they did not indicate what types of theories and approaches are needed to advance the science of positive psychology (Donaldson et al., 2021). Donaldson et al. (2021) also stated that they did not stipulate the criteria for what exactly constitutes a “positive theory” nor highlight the methods/processes required to generate knowledge in positive psychology.

Robbins and Friedman (2018), further argued that Seligman and Csikszentmihalyi (2000) regard Aristotle's virtue ethics as a fundamental building block of positive psychology, but ignore the philosophical foundation of his claims. While Aristotle described virtues as dynamic and working in unity, Seligman and Csikszentmihalyi (2000) see psychological strengths as working in isolation from each other. Robbins and Friedman (2018) proceed to argue that positive psychology is aimed at developing or at finding “goodness” or “the good life,” yet point out that Seligman and Csikszentmihalyi (2000) offer no clear vision as to what this may consist of and that the main assumptions of positive psychology rely on unexamined assumptions.

Donaldson et al. (2021) argued that positive psychology cannot collate a series of grand theories without a unifying metatheory to expand its theoretical value proposition. Grand theories are abstract theoretical assumptions that provide a means to formally organize or arrange knowledge of a particular concept or social phenomenon (Skinner, 1990). It provides a means through which positive psychological phenomena can be interpreted and explained (Donaldson et al., 2021). Critics argue that most grand theories currently underpinning positive psychology are “borrowed” from other paradigms such as social-, behavioral-, and cognitive psychology (e.g., Self-Determination Theory and Existentialism (Wong and Roy, 2017). The attempts of positive psychology to construct unique grand theories have been criticized and, in some cases, even disproven. For example, Fredrickson (2001) broaden-and-build theory is built on the assumption that only positive emotions broaden individuals' awareness which spirals them upwards toward creativity and performance. This assumption, however, stands in contrast to conventional wisdom in the science of emotions, which states that “negative emotions” (e.g., stress, frustration, unmet psychological needs) facilitate growth, motivation and performance and lead to creativity and resilience (Wong and Roy, 2017; Friedman and Brown, 2018). Gable and Harmon-Jones (2010) found that exposure to negative emotions increased the breadth of attention, focus, and drive. Other grand theories and elements that are central to positive psychology, such as Seligman's (2011) PERMA, Fredrickson and Losada's (2005) critical positivity ratio and the positive relationship science (Sheldon et al., 2010), have also been subjected to criticism.

To address these challenges, Robbins and Friedman (2018) suggested that the discipline clarifies its core values and philosophical foundation to move forward. Specifically, they mentioned that Positive Psychology should clarify its:

(a) metaphysical perspective of reality (e.g., How does positive psychology define reality? How do its values relate to what is known about reality?).
(b) epistemological beliefs (e.g., What is positive psychology's core values, and how do current ways of “knowing” such as its scientific methods help address problems associated with values. What values do positive psychological researchers hold regarding maintaining scientific integrity and the pursuit of unbiased truth? How do positive psychologists distinguish between competing theories? What criteria are used to determine what theories should be adopted or disregarded?).
(c) ethical position in theory development and practice (i.e., the moral and ethical decision-making process in theory building).

**Second**, the lack of metatheory leads *positive psychology to suffer from the “jingle- jangle” fallacy*. The *Jingle fallacy* occurs when different concepts or approaches within a discipline are erroneously assumed to be the same because of a shared name or label (Marsh, 1994). For example, “flourishing” is an essential concept within the positive psychological lexicon, yet three approaches to flourishing exist and are used interchangeably within the literature: Keyes (2002), Diener et al. (2010) and Seligman (2011). Diener et al. (2010) indicated that flourishing is based on the eudemonic tradition where people should feel good and function well. In essence, Diener et al. (2010) see flourishing as a measure of social-psychological prosperity. On the other hand, Keyes (2002) defined flourishing as a top-end human experience characterized by high levels of emotional-, psychological-, and social wellbeing. Finally, Seligman (2011) indicated that flourishing is a subjective, emotive experience characterized by high levels of positive emotions, engagement, relationships, meaning and achievement. There are clear conceptual distinctions between these three approaches, yet researchers erroneously use arguments from Keyes and Seligman to, for example, erroneously support the theoretical assumptions of Diener and vice versa (c.f. Nel, 2019 for an example). Various other positive psychological concepts ranging from engagement (cf. Kahn, 1990 vs. Schaufeli et al., 2006, vs. Seligman, 2011) to strengths (cf. Peterson and Seligman, 2004 vs. Rapp and Goscha, 2011) face the same problem.

Positive psychological constructs are also criticized for suffering from the “*Jangle Fallacy*”, where different terms are used to describe the same construct or where old psychological constructs are repackaged in new “jackets” to seem novel or new (Van Zyl et al., 2021). For example, “Joy” is seen as indistinguishable from other factors such as happiness, pleasure, positive emotion or the emotionality aspect of extraversion (Schnitker et al., 2020). Another example is “Psychological Capital” [PsyCap: Luthans (2002)], where four well-established theoretical concepts (hope, optimism, resilience and self-efficacy) were merely combined into a higher-order factor and presented as a new concept (Youssef-Morgan et al., 2022). Similarly, “Grit” (Duckworth et al., 2007) is seen one of the best predictors of personal success and performance, yet is indistinguishable from factors like conscientiousness or diligence (Van Zyl et al., 2021). Given that these factors are critiqued for not contributing anything “new,” it may further degrade the identity and credibility of the discipline. For science to progress, researchers should try to avoid the Jingle-Jangle fallacy when constructing new concepts or approaches in positive psychology (Schnitker et al., 2020).

**Third**, critics have *questioned the validity of positive psychological assessment measures*, aiming to assess positive states, traits, and behaviors (Wong and Roy, 2017). Van Zyl and Ten Klooster (2022) highlighted that popular positive assessment measures seem to produce different factorial structures, different levels of reliability and use questionable indicators for predictive validity in their validation processes. In addition, Lomas et al. (2021) and Van Zyl et al. (in review) have questioned the cultural fairness of positive psychological assessment measures. For example, the popular 12-item Grit-O Scale (Duckworth et al., 2007) has been shown to produce more than ten different factorial models in different studies ranging from a unidimensional model, to a bi-factor ESEM model with varying ranges of internal consistency (Van Zyl et al., 2020a). In a multi-national study, Van Zyl et al. (in review) further found that traditional confirmatory factor analytical models of the Grit-O scale were culturally biased.

Wong and Roy (2017) argued that this might be due to researchers employing “quick and dirty” approaches when developing new psychological measures, such as only using a single sample or not including a full range of concurrent and discriminant validity measures in the validation process. Therefore, given that measurement is at the core of any psychological science, researchers should be more rigorous in their approaches to developing and validating new measures. This challenge provides an opportunity for further investigation into the psychometric properties of current psychological assessments (incl. their cross-cultural relevance) and to develop more innovative approaches to the measurement of positive psychological states-, traits-, and behaviors.

**Fourth**, Wong and Roy (2017) argued *that positive psychological interventions fail to produce significant or sustainable changes* in participants' positive states, traits, and behaviors. Various meta-analyses and systematic literature reviews based on the effectiveness of positive psychological interventions have shown that most only produce small changes in wellbeing in the short term and that the long-term sustainability of such is highly questionable (Bolier et al., 2013; Ivandic et al., 2017; Donaldson et al., 2019; Roll et al., 2019). Applied to organizational contexts, Donaldson et al. (2019) found that most interventions only show small to marginal changes in important individual and organizational outcomes over the short and medium-term. Further, attempts at replicating the effects of popular positive psychological interventions have failed in various studies (Efendic and Van Zyl, 2019; Khanna and Singh, 2019; Mongrain and Anselmo-Matthews, 2019; Krifa et al., 2021). Mongrain and Anselmo-Matthews (2019) also argued that positive psychological interventions do not always produce unconditionally positive or beneficial results, whereas some interventions could cause harm. They argue that researchers fail to control for these potential negative consequences in their intervention designs (Mongrain and Anselmo-Matthews, 2019). For example, gratitude interventions can make people unhappy (Gulliford and Morgan, 2017) by increasing feelings of indebtedness to others, guilt, obligation and embarrassment (Watkins et al., 2006). These issues, along with the very sparse literature on the long-term effects of positive psychological interventions, pose interesting future research opportunities for intervention research.

**Fifth**, when hypotheses cannot be accepted or prior findings cannot be replicated, *positive psychologists rely on “contextual factors” for justification rather than self-correcting or updating existing theories* (Parks and Schueller, 2014; Friedman and Brown, 2018). For example, in recent job crafting intervention studies, no significant changes in the hypothesized outcome factors could be found (c.f. Demerouti et al., 2019; Hulshof et al., 2020). Here, the authors justified the findings by arguing that contextual factors within these organizations (such as organizational restructuring or the introduction of a new information management system) played a role in why these interventions were ineffective. These authors also argued that these organizational changes usually cause severe stress and anxiety, decreasing wellbeing/performance. As such, the authors argued that null findings imply that the intervention actually buffered against the environment's adverse effects on participants' wellbeing (Demerouti et al., 2019; Hulshof et al., 2020). As such, critics indicate that positive psychology is not self-correcting in nature, and unexpected or negative results (which differ from original expectations) are defended rather than explored and theories updated (Friedman and Brown, 2018; Hughes, 2018). This grand challenge provides opportunities for replication studies and highlights a need to measure and control for environmental factors within intervention studies.

**Sixth**, positive psychology is built on a belief in *empirical science and the complexity of statistical analysis to provide simple solutions to complex problems*. Friedman and Brown (2018) argued that the positive psychological community's infallible belief in the legitimacy of the analytical technique used to analyze the data withholds them from critiquing or scrutinizing empirical results. Further, Efendic and Van Zyl (2019) argued that the novelty of the contribution of a paper usually relies upon the statistical methodology used to analyze the data; where simple research questions are complicated with “advanced statistical techniques” to enhance its perceptive value or contribution to the discipline. There is thus an over-emphasis on quantification in positive psychology, where researchers erroneously believe that only rigorous (quantitative) methods are required to ensure scientific development (Friedman and Brown, 2018). Wong and Roy (2017) emphasize that positive psychology over-relies on scientism and the belief that the positivist paradigm is the only scientific approach to examining psychological phenomena. Lomas et al. (2021) suggested that researchers embrace robust qualitative and action research methods as well as employ more mixed-method approaches to answer important questions.

**Seventh**, critics argue that *positive psychology is culturally biased*. Positive psychology is criticized for being a primarily Western or European enterprise, where findings from Western, Educated, Industrialized, Rich and Democratic (WEIRD) contexts are generalized to the entire human population (Hendriks et al., 2019). Positive psychology is positioned as an “indigenous psychology that is universally applicable and relevant,” however it has neglected the cultural-, historical- and societal foundations underpinning the experience or development of positive states/traits/behaviors (Marecek and Christopher, 2018). In contrast to Seligman and Csikszentmihalyi (2000) position, critics argue that positive psychology is not objective nor value-neutral but rather prescriptive and directive in defining the elements of what a good life *should* constitute (Marecek and Christopher, 2018). Hendriks et al. (2019) argued that positive psychology's ethos is deeply rooted in the North-American ideology that the pursuit of happiness is a strongly individualistic process and negates the importance of indigenous knowledge. In other words, positive psychology is mainly individualistic in nature and positions the self as the center of the proverbial universe, where thoughts/feelings/behaviors are caused by internal processes and not influenced by environmental factors (Marecek and Christopher, 2018). However, numerous cross-cultural studies have shown that collectivistic and individualistic cultures view mental health and wellbeing concepts differently (Marecek and Christopher, 2018; Hendriks et al., 2019).

Unlike in individualistic cultures, those from collectivistic cultures tend to see wellbeing and mental health as a function of social contexts (e.g., family contexts, community wellbeing etc). Further, popular psychological assessment tools such as the aforementioned Grit-O scale and Mental Health Continuum-SF have shown not to be invariant across cultures; and that differences in interpretation of items lead to different factorial structures (Van Zyl and Olckers, 2019; Van Zyl and Ten Klooster, 2022). This, in itself, is worrisome because not only are there issues in the measurement of these constructs, but there could also then be differences in how each overall construct is seen/experienced/defined. Finally, popular positive psychological interventions built around WEIRD values may not be applicable to or useful for other non-WEIRD contexts (Van Zyl and Rothmann, 2020). For example, within South Africa, Van Zyl and Rothmann (2014) found a combination of popular self-administered intentional activities used in WEIRD contexts such as the gratitude visit, acts of kindness and the like, didn't result in changes in the associated a priori factors within a multi-cultural environment. Therefore, western orientated theories, assessment measures and interventions may not be applicable in other contexts. There is thus both a need and an opportunity for more indigenous positive psychological approaches and concepts to wellbeing. Further, this challenge also provides a foundation for more cross-cultural and cross-national studies on positive psychological concepts, methods and theories.

## Future Perspectives and Opportunities

Based on the abovementioned discussion, Positive Psychology faces various philosophical, methodological, and conceptual challenges. These grand challenges provide unique opportunities to expand the positive psychology discipline and to address societal issues through rigorous- and helpful insights from the discipline. With this sentiment in mind, we championed the launch of a new section dedicated to positive psychology in *Frontiers in Psychology*. With this new section, we aim to be an outlet for research to address these grand challenges and capitalize on their present opportunities. We, therefore, call upon the positive psychological fraternity to direct their attention to addressing these grand challenges and to expand the discipline through four broad focus areas aimed at:

- *Tackling wicked and ill-defined problems*, e.g., inequality, poverty, climate problems, and giving priority to society over individualism (Bentley and Toth, 2020) and the precarity in societies (Baart, 2021) that impact the good life. Multi-, inter- and transdisciplinary research and phenomenon-based learning (Lonka, 2018) are essential for solving wicked and ill-defined problems. It is imperative for positive psychologists to determine how they can retain breadth, diversity, and multidisciplinary thinking in an increasingly specialized world (Epstein, 2019).
- *Conducting studies in WEIRD and non-WEIRD contexts*. Western concerns may influence questions positive psychological researchers ask and the theories they develop (Gelfand et al., 2017). People outside developed countries face daily conflicts, terrorism, corruption, and poverty. It is critical that Positive Psychology poses new questions that reflect different societal values and assumptions and socio-political realities. Minorities and socioeconomically disadvantaged individuals are often missing in research, despite evidence that wellbeing and health are linked with sociodemographic factors (Ryff, 2022).
- *Integrating positive and negative experiences* (Ryff, 2022). For example, it is essential to study the co-activation of positive and negative emotions to understand how people master stressors and cope with them.
- *Building flourishing, sustainable, and “good” institutions, and societies*. In this regard, the individual, context, and interaction between them are critical. For example, the social-ecological resilience model (Ungar et al., 2020) and the sustainable employability model (Van der Klink, 2019) regard the interaction of individuals between individuals and contexts as critical for sustainable development outcomes. Research on “good organizations” (see www.goodorganisations.com) contributes to the mission of promoting organizations humane, socially responsible, and productive. In addition, understanding the dynamics of care and compassion for the self and others in different populations is vital (Ryff, 2022). Positive psychologists should urgently put their expertise to understand and improve individual wellbeing and community building (Poortinga, 2021).
- *Social justice, fairness and inclusion in institutions and societies* (Prillentensky and Prillentensky, 2021). In this regard, multidisciplinary and interdisciplinary approaches, such as the capability approach (CA; Nussbaum, 2011; Robeyns, 2017; Van der Klink, 2019), offer a framework for understanding the capabilities and functioning of people in different contexts.
- *Conceptualizing, constructing, and validating psychological measures applicable to cultural and disadvantaged groups* (Ryff, 2022). Studies about promising psychological methods are essential.

## Conclusion

Although it is beyond the scope of this grand challenge paper to reflect upon each of these criticisms and debate their scientific merit, we believe it is essential for the scientific community to develop solutions or responses to each of these challenges. We believe that these challenges provide exciting opportunities for the discipline to grow and develop into areas previously unknown. Therefore, we hope that this consolidated view as to some of the main challenges and opportunities for positive psychology may inspire researchers to build out the discipline and facilitate its development as a science.

## Author Contributions

LZ conceptualized and drafted the original manuscript. SR and LZ attended to the revisions. Both authors contributed equally to the final manuscript.

## Conflict of Interest

The authors declare that the research was conducted in the absence of any commercial or financial relationships that could be construed as a potential conflict of interest.

## Publisher's Note

All claims expressed in this article are solely those of the authors and do not necessarily represent those of their affiliated organizations, or those of the publisher, the editors and the reviewers. Any product that may be evaluated in this article, or claim that may be made by its manufacturer, is not guaranteed or endorsed by the publisher.
